# Investigating risk factors of hemorrhagic fever of renal syndrome (HFRS) in Qingdao, Shandong province, China

**DOI:** 10.1371/journal.pone.0338514

**Published:** 2025-12-15

**Authors:** Ying Li, Jing Jia, Runze Lu, Liyan Dong, Lizhu Fang, Litao Sun, Zongyi Zhang, Qing Duan, Lijie Zhang, Kunzheng Lv, Huilai Ma

**Affiliations:** 1 Chinese Field Epidemiology Training Program, Chinese Center for Disease Control and Prevention, Beijing, China; 2 National Institute for Viral Disease Control and Prevention, Chinese Center for Disease Control and Prevention, Beijing, China; 3 Qingdao Municipal Center for Disease Control and Prevention, Qingdao, Shandong Province, China; 4 Institute for Infectious Disease Control and Prevention, Shandong Center for Disease Control and Prevention, Jinan, Shandong Province, China; 5 Office of Epidemiology (Technical Guidance Office for Patriotic Health Work), Chinese Center for Disease Control and Prevention, Beijing, China; Gabriele d'Annunzio University of Chieti and Pescara: Universita degli Studi Gabriele d'Annunzio Chieti Pescara, ITALY

## Abstract

**Background:**

Qingdao, a historically high-risk area for hemorrhagic fever with renal syndrome (HFRS) in China, is undergoing agricultural mechanization and urbanization. However, the specific risk factors for HFRS in this context remain unclear. This study sought to determine the risk factors for HFRS in Qingdao.

**Methods:**

Community-based, 1:2 case-control study. Each case was matched with two healthy neighborhood controls based on biological sex, age, and the same neighborhood or village. Univariate and multivariate conditional logistic regression analyses were performed. Furthermore, stratified analyses were performed to explore risk factor heterogeneity between the peak season for Hantaan virus (HTNV) type HFRS (October-January) and other months.

**Results:**

93 cases (73.2%, 93/127) reported from January 2022 to September 2023 and 186 controls completed this questionnaire. Farmers accounted for the highest proportion (68.8%, 64/93). In multivariate logistic regression analysis, there were three significant risk factors for HFRS: piles of firewood and/or grain in residential yards (odds ratio [*OR*]=3.75, 95% *CI*: 2.14–6.55), mite and/or flea bites (*OR*=1.83, 95% *CI*: 1.06–3.18) and contacting with rats and/or their excreta (*OR*=1.73, 95% *CI*: 1.09–2.74); three variables represented significant protective factors for HFRS: frequency of sun exposure for quilts and bedding (*OR*=0.41, 95% *CI*: 0.19–0.90), rodent control measures at home (*OR*=0.50, 95% *CI*: 0.30–0.81) and knowing the main sources of HFRS transmission (*OR*=0.58, 95% *CI*: 0.36–0.90). Stratified analysis revealed that the influence of these factors varied by season, with rodent contact and control measures being particularly salient during the HTNV peak season.

**Conclusion:**

This study provides the first comprehensive evidence of risk and protective factors for HFRS in Qingdao, highlighting the role of rodent control, promoting comprehensive health education, environmental management, and personal protection. However, the results should be interpreted considering the study’s limitations, including a 73.2% response rate and the potential for recall bias.

## Introduction

Hemorrhagic fever with renal syndrome (HFRS), also known as epidemic hemorrhagic fever (EHF), is an acute interstitial nephropathy characterized by fever, headache, abdominal pain, renal dysfunction, and various hemorrhagic manifestations [1–4]. HFRS is predominantly endemic in Asia and Europe and seriously endemic in China, with 70–90% of the notified cases worldwide [5,6]. HFRS is a rodent-borne disease caused by hantaviruses in the Hantaviridae family within the order Bunyavirales [2,4,7]. The viruses that cause HFRS include Hantaan (HTNV), Dobrava, Saaremaa, Seoul (SEOV), and Puumala. HTNV carried by *Apodemus agrarius* mice and SEOV carried by *Rattus norvegicus* rats causes most HFRS in China [8]. There has been no targeted treatment for HFRS [9], and supportive therapy is the mainstay of care for patients with Hantavirus infections. Despite the fact that intensive measures have been implemented, HFRS remains a major public health problem in China [10].

Shandong province is one of the traditional sources of HFRS and is among the most serious endemic areas in China [11–13]. HFRS cases in Shandong province were first reported in 1968 [13]. The reported HFRS cases in Shandong province accounted for 11.0% of total cases in the whole country in 2004–2021 (24,592/2,243,960), which ranks fourth among the 31 provinces, municipalities, and autonomous regions in the country [10]. Qingdao is one of the key areas for HFRS in Shandong province. 2,220 HFRS cases were reported in Qingdao from 2010–2022, with an average annual incidence rate of 1.89/100,000, which is higher than the average for Shandong province. Qingdao has a relatively high HFRS case-fatality rate, with a cumulative total of 56 HFRS deaths reported in Qingdao from 2010–2022, a case-fatality rate of 2.5%. The HFRS case-fatality rate after 2012 is less than 1% in China [10], and the HFRS case-fatality rate from 2017–2020 is 1.5% in Shandong province [14]. The epidemic peak of HTNV-type HFRS (from October to January) was observed every year in Qingdao city, while the epidemic peak of SEOV-type HFRS (from March to June) was not obvious.

People are generally susceptible to Hantavirus, but the risk of infection depends mainly on exposure opportunities influenced by individual lifestyle, occupational habits, and environmental factors that affect human-rodent contact. The regional distribution of host animals can change with conditions such as food and temperature, and factors such as social development and economic activities also largely influence the ecological and habitat characteristics of rodent populations, which in turn affect the epidemiological pattern of HFRS. Case-control risk factor surveys for HFRS incidence have been conducted in some areas of China. The most recent ones in Shandong province were in Jining City in 2002 [15] and Linyi city in 1997 [16]. These studies suggest that risk factors for infection include direct contact with rodents, residential rodent infestation, exposure to rodent contaminants, living in a village-side household, cleaning long-abandoned sheds or houses, keeping pets in the home, and a history of field labor in the outbreak area. Vaccination is the most effective measure for individuals to prevent HFRS, and in China, HFRS immunization was included in the Expanded Programme on Immunization (EPI) starting in 2008, and routine immunization of people aged 16–60 years was conducted using high-incidence towns and villages in high-incidence provinces as the target population. In addition, raising residents’ awareness of HFRS, actively carrying out rodent prevention and extermination activities, and taking protective measures when engaging in high-risk behaviors can reduce the incidence of the disease.

Qingdao, as a city with a high incidence of HFRS in Shandong province [17], has never conducted a population-based study of the risk factors for the development of HFRS before and does not know much about the living environment and conditions of the residents, their knowledge of HFRS, whether they have carried out rodent prevention and rodent eradication activities, and their vaccination status. Qingdao has been experiencing rapid agricultural mechanization and urbanization. And the growth of the total power of agricultural machinery [18] and the development of urbanization [19] in Qingdao also affect the incidence of HFRS to a certain extent. Currently, the risk factors for HFRS in Qingdao are not clear and similar to those in other regions, and there needs to be a more scientific basis to prevent HFRS. The present study was designed to investigate the risk factors for HFRS in Qingdao, with the aim of providing a scientific basis for the development of effective and feasible interventions to reduce the incidence of HFRS in Qingdao further and alleviate the social and economic burden of HFRS in Qingdao, and to provide a reference for the study of similar naturally occurring epidemiologic diseases. In order to understand the possible risk factors of HFRS in Qingdao city in recent years, a community-based case-control study was conducted to identify the risk factors for HFRS and to supplement the previous studies.

## Methods

### Study area

Qingdao city is a tourist port city in Eastern China and is located on the west coast of the Pacific Ocean along the coast of the Yellow Sea, at the southern end of the Shandong Peninsula (35°35′-37°09′ N, 119°30′-121°00′ E). The land area of the jurisdiction is 11,282 km^2^, and the sea area is 12,240 km^2^. Qingdao is a hilly city along the seashore, with terrain that is high in the east, low in the west, raised in the north and south, and low in the center. Mountains, hills, plains, and depressions account for approximately 15.5%, 2.1%, 37.7%, and 21.7%, respectively, of the city’s total area. Qingdao belongs to the northern temperate maritime monsoon climate zone. Qingdao governs seven districts and three county-level cities. The resident population of Qingdao was 10,134,838 in 2022.

### Data and sample collection

Information on HFRS cases in Qingdao city from January 2022 to September 2023 was collected from the National Infectious Diseases Surveillance And Reporting System of the Chinese Center for Disease Control and Prevention (China CDC). The information about individual HFRS cases included age, occupation, disease onset, confirmation date, case category, residential address, and telephone number. Suspected patients with HFRS were diagnosed in accordance with the “Diagnostic Criteria for Epidemic Hemorrhagic Fever WS278-2008” issued by the Ministry of Health of China in 2008. Clinically diagnosed and laboratory-confirmed cases were included in our study. Ninety-three HFRS cases were investigated as cases, and 186 controls were recruited following the 1:2 matching method. The controls were randomly chosen among the people who lived in the same community or village, with an age gap of less than 5 years, and the same biological sex.

### Investigation of risk factors

A standardized questionnaire was used to investigate both cases and controls. Information about a month before the onset of the case (the ‘pre-morbidity’ in the control exposure history refers to the onset time of the case paired with it) was collected. The information includes general information (e.g., name, biological sex, age, primary occupation, phone number); incidence (e.g., onset time, place of onset, visiting time, diagnosed time); living environment (e.g., housing type, indoor hygiene; indoor rodent holes, indoor rodent population; surrounding environment; housing location; whether the housing was adjacent to ponds or rivers whether there were firewood piles around the housing; type of living floor; whether there were rodents in the workplace); having contact with rodents (Table 1).

**Table 1:** Results of univariate analysis of responses to a survey in a 1:2 case-control study of HFRS in Qingdao city.

Variables		Cases	Controls	*P*	OR (95% *CI*)
		n (%)	n (%)		
Occupation	Farmer	64 (68.8)	118 (63.4)	0.468	1.18 (0.76-1.82)
	Non-farmer	29 (31.2)	68 (36.6)		
Educational background	Uneducated	12 (12.9)	11 (5.9)	0.081	1.86 (0.93-3.74)
	Primary or middle school	58 (62.4)	116 (62.4)	0.484	1.19 (0.73-1.93)
	High School and above	23 (24.7)	59 (31.7)		
Having underlying diseases	Yes	16 (17.2)	43 (23.1)	0.353	0.75 (0.45-1.33)
	No	77 (82.8)	143 (76.9)		
Average income (RMB)	≤20,000	55 (59.1)	98 (52.7)	0.563	1.14 (0.73-1.76)
	>20,000	38 (40.9)	88 (47.3)		
Knowing the pathogen of HFRS	Yes	18 (19.4)	88 (47.3)	0.000	0.39 (0.23-0.66)
	No	75 (80.6)	98 (52.7)		
Knowing the main sources of HFRS transmission	Yes	39 (41.9)	145 (78.0)	0.000	0.37 (0.25-0.56)
	No	54 (58.1)	41 (22.0)		
Knowing the most vulnerable organs of HFRS	Yes	23 (24.7)	86 (46.2)	0.005	0.51 (0.32-0.82)
	No	70 (75.3)	100 (53.8)		
Housing location (by or outside the village)	Yes	54 (58.1)	60 (32.3)	0.001	2.00 (1.33-3.03)
	No	39 (41.9)	126 (67.7)		
House near pondgully	Yes	53 (57.0)	26 (14.0)	0.000	3.35 (2.23-5.06)
	No	40 (43.0)	160 (86.0)		
Frequency of house cleaning	Never	3 (3.2)	0 (0)	0.035	3.55 (1.10-11.51)
	Occasionally	52 (55.9)	89 (47.8)	0.206	1.31 (0.86-1.99)
	Often	38 (40.9)	97 (52.2)		
Sprinkling while cleaning house	Yes	38 (40.9)	108 (58.1)	0.028	0.63 (0.42-0.95)
	No	55 (59.1)	78 (41.9)		
Wearing a mask while cleaning house	Yes	2 (2.2)	40 (21.5)	0.004	0.12 (0.03-0.50)
	No	91 (97.8)	146 (78.5)		
Spare rooms at home	Yes	48 (51.6)	79 (42.5)	0.239	1.28 (0.85-1.92)
	No	45 (48.4)	107 (57.5)		
Presence of rodent holes at home	Yes	9 (9.7)	14 (7.5)	0.616	1.19 (0.60-2.37)
	No	84 (90.3)	172 (92.5)		
Presence of rats at home	Yes	55 (59.1)	39 (21.0)	0.000	2.85 (1.88-4.31)
	No	38 (40.9)	147 (79.0)		
Rodent control measures at home	Yes	24 (25.8)	82 (44.1)	0.017	0.57 (0.36-0.90)
	No	69 (74.2)	104 (55.9)		
Raising livestock and/or poultry	Yes	36 (38.7)	41 (22.0)	0.018	1.66 (1.09-2.52)
	No	57 (61.3)	145 (78.0)		
Piles of firewood and/or grain in residential yards	Yes	76 (81.7)	54 (29.0)	0.000	5.12 (3.03-8.67)
	No	17 (18.3)	132 (71.0)		
Frequency of sun exposure for quilts and bedding	Yes	7 (7.5)	73 (39.2)	0.000	0.20 (0.09-0.44)
	No	86 (92.5)	113 (60.8)		
Storage of rice, white flour and other dry rations	Covered cylinder	21 (22.6)	119 (64.0)	0.027	0.30 (0.10-0.87)
	Uncovered cylinder	1 (1.1)	0 (0)	0.535	2.00 (0.22-17.89)
	Cloth or snake skin bag	67 (72.0)	63 (33.9)	0.953	1.03 (0.38-2.83)
	No storage	4 (4.3)	4 (2.2)		
Storing leftovers with protection	Yes	73 (78.5)	181 (97.3)	0.000	0.36 (0.22-0.59)
	No	20 (21.5)	5 (2.7)		
Heating leftovers before eating	Never	0 (0)	11 (5.9)	0.954	0.00 (0.00-7.53E + 176)
	Occasionally	16 (17.2)	44 (23.7)	0.451	0.62 (0.18-2.14)
	Often	74 (79.6)	127 (68.3)	0.796	0.86 (0.27-2.73)
	No leftovers	3 (3.2)	4 (2.2)		
HFRS vaccination	Yes	0 (0)	25 (13.4)	0.049	0.04 (0.00-0.99)
	No	93 (100.0)	161 (86.6)		
Hand-washing habits before meals	Yes	74 (79.6)	181 (97.3)	0.289	0.58 (0.21-1.59)
	Not necessarily	15 (16.1)	1 (0.5)	0.264	1.88 (0.62-5.65)
	No	4 (4.3)	4 (2.2)		
Breaks in the skin	Yes	9 (9.7)	6 (3.2)	0.071	1.89 (0.95-3.70)
	No	84 (90.3)	180 (96.8)		
Breach in the oral cavity	Yes	1 (1.1)	0 (0)	0.271	3.02 (0.42-21.68)
	No	92 (98.9)	186 (100.0)		
Mite and/or flea bites	Yes	18 (19.4)	6 (3.2)	0.000	2.55 (1.53-4.27)
	No	75 (80.6)	180 (96.8)		
Contacting with rats and/or their excreta	Yes	34 (36.6)	2 (1.1)	0.000	3.89 (2.55-5.93)
	No	59 (63.4)	184 (98.9)		

Abbreviations: CI confidence interval, HFRS hemorrhagic fever with renal syndrome, OR odds ratio.

### Statistical analysis

The risk factors of exposure were analyzed by univariate analysis and multivariate logistic regressions. Chi-square test, *t*-test, and univariate and multivariate conditional logistic regression analysis were performed with SPSS version 20.0 (the Statistical Product and Service Solutions, Chicago, IL, USA). The odds ratios (*OR*s) with 95% confidence intervals (*CI*s) were used to quantify the association strength between variables. Variables with *P*-value ≤ 0.10 in the univariate analysis were included in the multivariate regression model. The backward stepwise elimination procedure was applied to exclude the variables with *P*-value > 0.05 in the multivariate regression model.

### Ethics considerations

Ethics and approval were sought and granted by the Shandong Center for Disease Control and Prevention (Approval for ethical review of biomedical research involving humans, ETH-2017020), and the written informed consent of all the participants. 93 cases reported between January 2022 and September 2023 and their matched controls participated in and completed this survey. Participation of subjects was voluntary, and written consent was obtained from all participants prior to data collection.

## Results

### Time and population distribution characteristics

**Fig 1:**
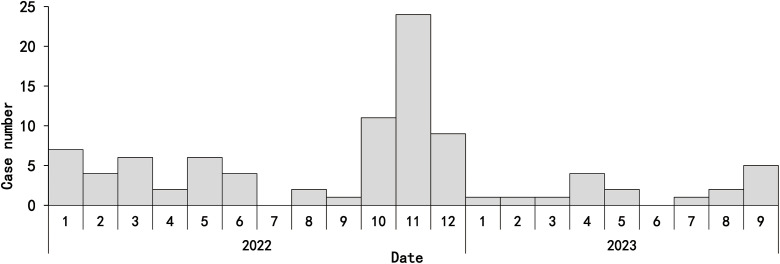
Monthly distribution of the surveyed HFRS cases in Qingdao City from January 2022 to September 2023.

**Fig 2:**
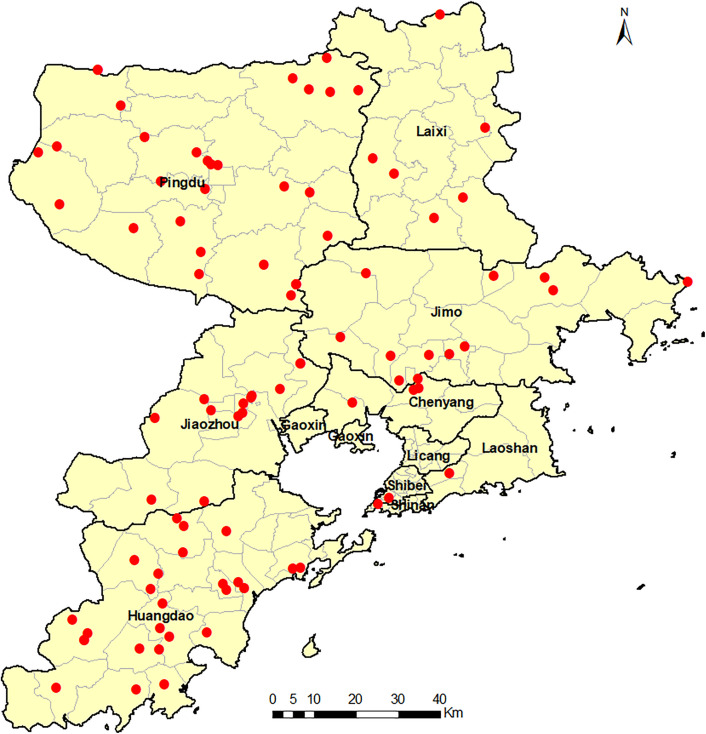
Distribution of the surveyed HFRS cases in Qingdao City from January 2022 to September 2023.

A total of 127 cases were reported from January 2022 to September 2023 (99 cases in 2022 and 28 cases in January-September 2023), of which one was a fatal case (2022). 93 cases (Figs 1 and 2) completed this questionnaire, with a response rate of 73.2% (93/127); the remaining 34 cases (26.8%, 34/127) were lost to follow-up, containing four fatal cases (the one reported in 2022 and three post-discharge deaths due to other reasons).

This study had 279 participants, including 93 cases and 186 matched controls. For the cases and controls, the mean ages were (55.2 ± 14.3) and (55.2 ± 13.7) years, respectively, and the difference was not statistically significant (*t *= −0.015, *P* = 0. 988). There were 210 (75.27%) males and 69 (24.73%) females, respectively. Of the 93 cases, 76 cases (81.7%, 76/93) were reported in 2022, and 17 cases (18.3%, 17/93) were reported in 2023, of which 47.3% (44/93) were reported in October-December 2022. Farmers accounted for the highest proportion (68.8%, 64/93), of which 37.6% (35/93) were pure farmers, and 31.2% (29/93) were “farmers + others”.

### Univariate analysis of and multivariate logistic regression analysis of risk factors

In the univariate conditional logistic regression model, there were 16 variables with statistical significance (*P* < 0.05); the other 12 variables were not significantly different between cases and controls (Table 1). Multivariate conditional logistic regression analysis was carried out for the remaining 16 variables screened out from univariate analysis. Variables were screened by the Wald forward method. The criteria for entering the equation was *P* < 0.05, and for eliminating the equation was *P* > 0.10. Six significant factors were identified, including three significant risk factors for HFRS: piles of firewood and/or grain in residential yards (*OR*=3.75, 95% *CI*: 2.14–6.55), mite and/or flea bites (*OR*=1.83, 95% *CI*: 1.06–3.18) and contacting with rats and/or their excreta (*OR*=1.73, 95% *CI*: 1.09–2.74); three variables represented significant protective factors for HFRS: frequency of sun exposure for quilts and bedding (*OR*=0.41, 95% *CI*: 0.19–0.90), rodent control measures at home (*OR*=0.50, 95% *CI*: 0.30–0.81) and knowing the main sources of HFRS transmission (*OR*=0.58, 95% *CI*: 0.38–0.90) (Table 2).

**Table 2:** Results of multivariate logistic regression analyses of potential risk factors for HFRS in Qingdao city.

Variables	*β*	*Wald*	*P*	*OR*	95% *CI*
Piles of firewood and/or grain in residential yards	1.321	21.521	0.000	3.75	2.14-6.55
Mite and/or flea bites	0.605	4.631	0.031	1.83	1.06-3.18
Contacting with rats and/or their excreta	0.546	5.357	0.021	1.73	1.09-2.74
Frequency of sun exposure for quilts and bedding	−0.894	4.882	0.027	0.41	0.19-0.90
Rodent control measures at home	−0.700	7.777	0.005	0.50	0.30-0.81
Knowing the main sources of HFRS transmission	−0.545	6.051	0.014	0.58	0.38-0.90

Abbreviations: CI confidence interval, HFRS hemorrhagic fever with renal syndrome, OR odds ratio.

### Stratified analysis by months of HTNV-type HFRS (from October to January) and other months

The epidemic peak of HTNV-type HFRS (from October to January) was observed every year in Qingdao city, while the epidemic peak of SEOV-type HFRS (from March to June) was not obvious. Thus, stratified analyses were conducted in this study to explore the difference in risk factors between cases in months of epidemic peak of HTNV-type HFRS and cases in other months (February to September).

In months of the epidemic peak of HTNV-type HFRS, there were 12 variables with statistical significance (*P *< 0.05); the other 16 variables were not significantly different between cases and controls (Table 3). Multivariate logistic regression analysis was carried out for the remaining 12 variables screened out from univariate analysis. Four significant factors were identified, including piles of firewood and/or grain in residential yards (*OR*=6.12, 95% *CI*: 2.69–13.93), contact with rats and/or their excreta (*OR*=1.99, 95% *CI*: 1.12–3.54), knowing the pathogen of HFRS (*OR*=0.44, 95% *CI*: 0.21–0.92), rodent control measures at home (*OR*=0.49, 95% *CI*: 0.26–0.92) (Table 4).

**Table 3:** Results of univariate analysis of responses to a survey in a 1:2 case-control study of HFRS in Qingdao city during the months with peak HTNV-type HFRS.

Variables		Cases	Controls	*P*	OR (95% *CI*)
		n (%)	n (%)		
Knowing the pathogen of HFRS	Yes	9 (17.3)	52 (50.0)	0.002	0.33 (0.16-0.67)
	No	43 (82.7)	52 (50.0)		
Knowing the main sources of HFRS transmission	Yes	21 (40.4)	77 (74.0)	0.001	0.40 (0.23-0.70)
	No	31 (59.6)	27 (26.0)		
Housing location (by or outside the village)	Yes	31 (59.6)	38 (36.5)	0.028	1.86 (1.07-3.24)
	No	21 (40.4)	66 (63.5)		
House near pondgully	Yes	33 (63.5)	13 (12.5)	0.000	4.15 (2.36-7.30)
	No	19 (36.5)	91 (87.5)		
Wearing a mask while cleaning house	Yes	1 (1.9)	27 (26.0)	0.017	0.09 (0.01-0.65)
	No	51 (98.1)	77 (74.0)		
Presence of rats at home	Yes	37 (71.2)	24 (23.1)	0.000	3.84 (2.11-7.00)
	No	15 (28.8)	80 (76.9)		
Rodent control measures at home	Yes	13 (25.0)	51 (49.0)	0.022	0.48 (0.26-0.90)
	No	39 (75.0)	53 (51.0)		
Piles of firewood and/or grain in residential yards	Yes	45 (86.5)	29 (27.9)	0.000	7.12 (3.21-15.80)
	No	7 (13.5)	75 (72.1)		
Frequency of sun exposure for quilts and bedding	Yes	5 (9.6)	39 (37.5)	0.005	0.27 (0.11-0.68)
	No	47 (90.4)	65 (62.5)		
Storing leftovers with protection	Yes	38 (73.1)	101 (97.1)	0.000	0.33 (0.18-0.61)
	No	14 (26.9)	3 (2.9)		
HFRS vaccination	Yes	0 (0)	14 (13.5)	0.141	0.04 (0.00-2.83)
	No	52 (100.0)	90 (86.5)		
Contacting with rats and/or their excreta	Yes	21 (40.4)	1 (1.0)	0.000	4.13 (2.37-7.18)
	No	31 (59.6)	103 (99.0)		

Abbreviations: CI confidence interval, HFRS hemorrhagic fever with renal syndrome, OR odds ratio.

**Table 4:** Multivariate logistic regression analyses of potential risk factors in months of peak of HTNV-type HFRS.

Variables	*β*	*Wald*	*P*	*OR*	95% *CI*
Piles of firewood and/or grain in residential yards	1.811	18.611	0.000	6.12	2.69-13.93
Contacting with rats and/or their excreta	0.690	5.533	0.019	1.99	1.12-3.54
Knowing the pathogen of HFRS	−0.823	4.832	0.028	0.44	0.21-0.92
Rodent control measures at home	−0.721	4.846	0.028	0.49	0.26-0.92

Abbreviations: CI confidence interval, HFRS hemorrhagic fever with renal syndrome, OR odds ratio.

In other months, there were 12 variables with statistical significance (*P* < 0.05); the other 16 variables were not significantly different between cases and controls (Table 5). Multivariate logistic regression analysis was carried out for the remaining 12 variables screened out from univariate analysis. Three significant factors were identified, including piles of firewood and/or grain in residential yards (*OR*=2.33, 95% *CI*: 1.10–4.96), frequency of sun exposure for quilts and bedding (*OR*=0.20, 95% *CI*: 0.05–0.85), knowing the main sources of HFRS transmission (*OR*=0.43, 95% *CI*: 0.23–0.81) (Table 6).

**Table 5:** Results of univariate analysis of responses to a survey in a 1:2 case-control study of HFRS in Qingdao city in non-peak months.

Variables		Cases	Controls	*P*	OR (95% *CI*)
		n (%)	n (%)		
Knowing the main sources of HFRS transmission	Yes	18 (43.9)	68 (82.9)	0.001	0.34 (0.18-0.62)
	No	23 (56.1)	14 (17.1)		
Knowing the most vulnerable organs of HFRS	Yes	9 (22.0)	39 (47.6)	0.029	0.44 (0.21-0.92)
	No	32 (78.0)	43 (52.4)		
Housing location (by or outside the village)	Yes	23 (56.1)	22 (26.8)	0.012	2.22 (1.20-4.10)
	No	18 (43.9)	60 (73.2)		
House near pondgully	Yes	20 (48.8)	13 (15.9)	0.002	2.60 (1.41-4.79)
	No	21 (51.2)	69 (84.1)		
Presence of rats at home	Yes	18 (43.9)	15 (18.3)	0.016	2.13 (1.15-3.96)
	No	23 (56.1)	67 (81.7)		
Raising livestock and/or poultry	Yes	17 (41.5)	12 (15.6)	0.009	2.30 (1.23-4.27)
	No	24 (58.5)	70 (85.4)		
Piles of firewood and/or grain in residential yards	Yes	31 (75.6)	25 (30.5)	0.000	3.71 (1.82-7.57)
	No	10 (24.4)	57 (69.5)		
Frequency of sun exposure for quilts and bedding	Yes	2 (4.9)	34 (41.5)	0.004	0.12 (0.03-0.51)
	No	39 (95.1)	48 (58.5)		
Storing leftovers with protection	Yes	35 (85.4)	80 (97.6)	0.041	0.41 (0.17-0.97)
	No	6 (14.6)	2 (2.4)		
Breaks in the skin	Yes	7 (17.1)	2 (2.4)	0.021	2.61 (1.16-5.88)
	No	34 (82.9)	80 (97.6)		
Mite and/or flea bites	Yes	13 (31.7)	3 (3.7)	0.001	3.11 (1.61-5.99)
	No	28 (68.3)	79 (96.3)		
Contacting with rats and/or their excreta	Yes	13 (31.7)	1 (1.2)	0.000	3.62 (1.87-6.98)
	No	28 (68.3)	81 (98.8)		

Abbreviations: CI confidence interval, HFRS hemorrhagic fever with renal syndrome, OR odds ratio.

**Table 6:** Multivariate logistic regression analyses of potential risk factors in non-peak months.

Variables	*β*	*Wald*	*P*	*OR*	95% *CI*
Piles of firewood and/or grain in residential yards	0.846	4.818	0.028	2.33	1.10-4.96
Frequency of sun exposure for quilts and bedding	−1.600	4.748	0.029	0.20	0.05-0.85
Knowing the main sources of HFRS transmission	−0.836	6.924	0.009	0.43	0.23-0.81

Abbreviations: CI confidence interval, HFRS hemorrhagic fever with renal syndrome, OR odds ratio.

## Discussion

This is the first case-control study of HFRS in Qingdao. The study included 73% of all cases of HFRS notified over a 21-month period, and included cases were distributed in nine of the ten counties and districts in the city (Figs 1 and 2). The present study was a 1:2 matched case-control study using multivariate conditional logistic regression analysis, which can better exclude the interference of confounding factors, reduce bias, and obtain reliable survey results. The case and control groups in the present study were balanced and consistent in terms of age, gender, and living conditions, which maximized the authenticity and reliability of the results.

Qingdao city is a high-prevalence area of mixed HFRS with a predominance of HTNV-type HFRS. Of the 28 factors investigated in Qingdao city in this study, 16 factors were associated with the onset of HFRS after univariate conditional logistic regression analysis. After multivariate conditional logistic regression analysis to eliminate possible confounding effects, three factors, such as piles of firewood and/or grain in residential yards (*OR*=3.747, 95% *CI*: 2.144–6.547), mite and/or flea bites (*OR*=1.831, 95% *CI*: 1.055–3.175) and contacting with rats and/or their excreta (*OR*=1.726, 95% *CI*: 1.087–2.739) were the probable risk factors for the onset of HFRS; whereas frequency of sun exposure for quilts and bedding (*OR*=0.409, 95% *CI*: 0.185–0.904), rodent control measures at home (*OR*=0.496, 95% *CI*: 0.303–0.812) and knowing the main sources of HFRS transmission (*OR*=0.580, 95% *CI*: 0.375–0.895) were the protective factors for the development of HFRS.

Hantaviruses are carried and transmitted by rodents, and rodents are the main source of HFRS. Direct contact with rodents or their feces is a direct exposure to the source of infection and carries a high risk of HFRS. In this study, contacting with rats and/or their excreta within one month before the onset of HFRS was a risk factor for the development of HFRS, in which direct contact with rodents as a risk factor for the development of HFRS was consistent with the results of previous studies (Xuancheng, Anhui province, 2020 [20]; Lianyungang, Jiangsu province, 1993 [21] and 2003 [22]; Jilin province, 2016 [23]; Jiande, Zhejiang province, 1992 [24]; and Shanghai, 1987 [25]), and exposure to rodent excreta as a risk factor for the development of HFRS was consistent with the results of the previous studies (Sishui county, Jining, Shandong province, 2002 [15]; Xi’an, Shaanxi province, 2013 [26] and 1990 [27]; Yanyuan county, Liangshan Yi Autonomous Prefecture, Sichuan province, 2009 [28]; Xiangshan county, Ningbo, Zhejiang province, 2010 [29]; Huaibin county, Xinyang, Henan province, 1997 [30]; and Neihuang county, Anyang, Henan province, 2002 [31]). In this study, rodent control measures at home were a protective factor for the development of HFRS, which was consistent with the results of the studies in Sishui county, Jining, Shandong province, in 2002 [15] and Guangzhou city, Guangdong province, in 2014 [32]. These findings are further supported by local rodent surveillance data. A longitudinal study in Qingdao (2010–2022) identified Rattus norvegicus as the predominant rodent species and confirmed the circulation of both HTNV and SEOV viruses, with a notable virus carriage rate detected in this species [17]. Besides, rodent control measures in the home were identified to be a protective factor during the months of peak HTNV-type HFRS, which may be related to the anti-rodent activities carried out during the Qingdao Patriotic Health Campaign in November every year. These findings suggest that individuals engaged in rodent eradication work or any other activity that involves contact with rats in areas impacted by HFRS should exercise caution to avoid exposure.

In this study, piles of firewood and/or grain in residential yards were identified as risk factors for the onset of HFRS, and this is consistent with the findings of the studies that found that the presence of grain straw in the yard of Junan county, Shandong province, in 1997 [16], and the presence of firewood piles or straw piles at home in Yanyuan county, Sichuan province, in 2009 [28]. The observed association may be attributed to two main reasons. First, many rural residents in Qingdao continue to use firewood for cooking, which is often stored permanently in yards. Second, such stacks provide preferred habitats for rodents and tend to accumulate large amounts of rodent excreta containing virus, thereby facilitating transmission via the aerosol route. To reduce rodent attraction and minimize human exposure, it is recommended to store firewood and grain in sealed containers or on elevated platforms positioned away from house walls, and to bring indoors only small amounts required for immediate use. Additionally, measures promoting the safe storage of firewood and grains—such as the use of rodent-proof containers or structures—should be encouraged.

The main and well-established mode of transmission of HFRS is through contact with host rodents carrying the virus or their excreta (feces, urine, saliva), but hypotheses of vectorial (mite-borne) and vertical transmission of HFRS has also been proposed [33]. As early as 1991, Song Gan’s study concluded that HFRS could be transmitted by mite vector, and Dong Bijun et al. concluded that mites carrying the virus could proliferate in the body and play a certain role as a vector in the transmission of Hantavirus [34]. As for the transmission of Hantavirus by fleas, Dong Bijun et al. concluded that they only carry the virus mechanically [34]. In our study, the finding that mite and/or flea bites within one month before disease onset was a risk factor for HFRS is consistent with the results of the 2020 study in Xuancheng city, Anhui province [20], in which insect bites were a risk factor for the development of HFRS. However, this observation should be interpreted cautiously. It is plausible that this association reflects confounding by rodent exposure, as mite/flea infestations are strongly linked to rodent presence. Our stratified analysis showed that contact with rats and/or their excreta was associated with mite/flea bites, supporting the idea that these arthropods may be indicators of rodent activity rather than independent transmission vectors. Therefore, we present this finding as hypothesis-generating, highlighting a potential indirect pathway worthy of further investigation, without challenging the established consensus of rodent-to-human transmission as the principal route.

Hantavirus infection is associated with daily lifestyle and hygiene in the household. This study also identified that frequent exposure for quilts and bedding to sunlight protects against HFRS. Stratified analysis similarly demonstrated that frequent sun exposure of bedding during other months (February to September) provides protective effects. The efficacy of this practice may be attributed to the antiviral effects of solar ultraviolet (UV) radiation. This is supported by experimental data showing that UV irradiation of HTNV for 3 minutes results in a greater than 5-log reduction in viral infectivity [35]. Furthermore, frequent airing of bedding may reflect better household hygiene, which not only reduces the ability of house mites to survive and reproduce but also inhibits their transmission. Therefore, strengthening the monitoring of inter-rodent epidemics and vigorous rodent eradication programs may be more effective in preventing HFRS by actively improving environmental sanitation conditions, increasing health education, and raising the level of hygiene among the general population.

A noteworthy finding of our study is the identification of “knowing the main sources of HFRS transmission” as a significant independent protective factor. This underscores the critical role of public health awareness in disease prevention. The higher level of knowledge among controls may reflect the success of existing and prior health education campaigns conducted in Qingdao. It suggests that individuals who are informed about the primary role of rodents in transmission are more likely to implement proactive measures to avoid exposure, such as improved food storage and rodent control. Consequently, our results provide strong empirical support for intensifying targeted health education as a feasible and cost-effective public health intervention. Future efforts should focus on reinforcing this knowledge, particularly in high-risk rural communities, to further reduce HFRS incidence.

Based on this study, we propose the following specific public health interventions to reduce hantavirus exposure and HFRS incidence: (1) Targeted Environmental Management: Launch “Clean Courtyard” initiatives in high-risk villages, focusing on the proper storage of firewood and grain. Promote the use of rodent-proof containers or elevated storage platforms away from dwelling walls. Encourage residents to store only small quantities for immediate use. (2) Enhanced and Precision Rodent Control: Reinforce the annual centralized rodent control campaign before the HTNV peak season. Establish a community-based rodent surveillance and reporting system where residents can report increased rodent activity for prompt response by public health teams. (3) Precision Health Education: Develop targeted messaging centered on the core concept that “rodents are the primary source of infection.” Use local examples and multiple channels (e.g., village broadcasts, social media) to educate residents on avoiding contact with rodents/excreta and using personal protective equipment during clean-up of potentially contaminated areas. (4) Promotion of Protective Behaviors: Actively promote the health benefits of regularly sun-drying quilts and bedding, especially during the humid non-peak seasons, as a simple and effective protective measure.

This study has some limitations. First, the case-control design is inherently susceptible to recall bias, which may have been exacerbated by the one-year recall period for some cases from 2022 and the potential for differential recall between cases and controls, possibly leading to an overestimation of the associations. Second, the 73.2% response rate, with data missing from 26.8% of eligible cases (including fatal cases), may introduce selection bias and limit the generalizability of our findings. Third, despite matching and multivariate adjustment, residual confounding from unmeasured factors, such as detailed agricultural practices, cannot be ruled out. Finally, the variable “firewood and/or grain piles” could not distinguish between these two materials, and despite using a structured variable selection process, our multivariate model may still be prone to overfitting, underscoring the need for external validation in future studies.

In summary, our findings indicate that firewood and/or grain stacks in residential yards, mite and/or flea bites, and contact with rats and/or their excreta are probable risk factors for HFRS in Qingdao and that frequent exposure of quilts and bedding to sunlight, rodent control measures in the home, and awareness of the main sources of HFRS transmission are likely protective factors. Targeted strategies, including improved rodent control, better health education, and behavior change, should be implemented among high-risk populations to reduce the incidence of HFRS.

## Supporting information

S1 Table Supporting Information file S1_Data.xlsx. This is the minimal data set required to replicate the findings of this study. This file contains the underlying data for all analyses.
(XLSX)
